# Home delivery of medications: Community pharmacists' perspectives on the pros and cons of the service

**DOI:** 10.3389/fpubh.2022.966145

**Published:** 2022-08-24

**Authors:** Rana Abu-Farha, Karem H. Alzoubi, Rama Alkhawaldeh, Rania Itani, Samar Karout, Tareq Mukattash, Eman Alefishat

**Affiliations:** ^1^Department of Clinical Pharmacy and Therapeutics, Faculty of Pharmacy, Applied Science Private University, Amman, Jordan; ^2^Department of Pharmacy Practice and Pharmacotherapeutics, University of Sharjah, Sharjah, United Arab Emirates; ^3^Department of Clinical Pharmacy, Faculty of Pharmacy, Jordan University of Science and Technology, Irbid, Jordan; ^4^Pharmacy Practice Department, Faculty of Pharmacy, Beirut Arab University, Beirut, Lebanon; ^5^Department of Pharmacology, College of Medicine and Health Science, Khalifa University of Science and Technology, Abu Dhabi, United Arab Emirates; ^6^Center for Biotechnology, Khalifa University of Science and Technology, Abu Dhabi, United Arab Emirates; ^7^Department Biopharmaceutics and Clinical Pharmacy, Faculty of Pharmacy, The University of Jordan, Amman, Jordan

**Keywords:** home delivery of medication, perception, willingness, community pharmacist, Jordan

## Abstract

**Objectives:**

The main goal of the current study was to investigate pharmacists' perception of home delivery of medications service in Jordan and their willingness to use the service.

**Method:**

This cross-sectional observational study was conducted between March and April 2022. The study targeted community pharmacists working at different community pharmacies across Jordan. The study questionnaire was distributed through Facebook to target Jordanian community pharmacists' groups.

**Results:**

Three hundred and twenty-four community pharmacists participated in the study, 75% (*n* = 244) of pharmacists reported being willing to use the home delivery and 274 (84.6%) thought it increases the efficiency of their community pharmacies' services. Only 129 (39.8%) pharmacists agreed or strongly agreed that unlike in-store service, home delivery of medications is suitable only for OTC but not for prescriptions medications Nearly half the number of participating pharmacists (*n* = 153, 47.2%) believe that the service is suitable for refill prescriptions but not for new prescriptions. Pharmacists believe that the foremost pros of the service were to continue life-saving medical treatment (*n* = 249, 76.9%), serve sick, elderly, and disabled patients (*n* = 241, 74.4%), and decrease congestion at health facilities (*n* = 228, 70.4%). On the other hand, the cons of this service, as perceived by pharmacists included failing to build a professional relationship with patients (*n* = 203, 62.7%), and the contribution to communication errors (*n* = 147, 45.4%). Logistic regression showed that pharmacists who serve 50 patients or more per day were more willing to use the service than those serving less than 50 patients per day (OR = 2.058, *P* = 0.032).

**Conclusion:**

The majority of participating pharmacists in this study were willing to use the service at their community pharmacies, especially those serving a large number of patients per day which may indicate the potential of this service in relieving the pressure on community pharmacies and allowing them to serve more patients efficiently.

## Introduction

During the COVID-19 pandemic, home delivery of medication service was one of the pharmacist's responsibilities, such services aimed to reduce disease transmission and spread *via* reducing congestion at health care facilities during the pandemic (1). This service was established in both institutional and community pharmacies (2). The service was provided by qualified pharmacists who prepare the medications with the right labeling and packaging requirements and deliver them to the verified address, patients were not charged any delivery fees (3). This approach is reportedly beneficial to minimizing patients' visits to hospitals and pharmacies when they are unable to attend due to mobility limitations, as well as for patients with multiple comorbidities. Moreover, home delivery of medication service saves patients time and money, increases patients' adherence to chronic medications, and it is more convenient for parents with children at home (1, 3).

One major challenge that may result after the implementation of the home delivery of medications service is the loss of face-to-face counseling with the pharmacist (3). There were also concerns about the ability to implement this service for all medications, as it could be suitable for refilling prescriptions but not recommended for new prescribed drugs, controlled substances, and temperature-controlled medications (4). Several studies in the literature have evaluated patients' perceptions of home delivery of medication services (4–6), but there is currently no information available about pharmacists' perceptions of this service. This necessitates a study to investigate pharmacists' perceptions of home delivery of medication services. So, the primary goal of the current study was to investigate pharmacists' perception of home delivery of medications service in Jordan and their willingness to use the service. Factors associated with pharmacists' willingness to use the service are also investigated.

## Methods

### Study design and participants

This is a cross-sectional observational study performed between March and April 2022. We conducted an online survey to evaluate community pharmacists' perception of home delivery of medication service that was started to be implemented partially in Jordan. The survey targeted community pharmacists working at different community pharmacies in different governorates across Jordan. The ethical standards defined by the World Medical Association Declaration of Helsinki guideline were followed in this study (7). The study was granted ethical approval by the institutional board committee at Applied Science Private University (Approval number 2022-PHA-4).

### Questionnaire development and data collection

The structured survey was adopted from a previous study that has evaluated pharmacists perception toward drive-thru service (8). Content and face validation were conducted by three experienced coauthors in the field. According to their feedback, the survey was amended and the final version had five main focused parts: (1) demographic characteristics, (2) questions to assess participants' general awareness and perception of home delivery of medication service, (3) statements to evaluate their perception toward the difference between home delivery of medication service and in-store refill of medications, (4) statements to assess participant perceptions toward the pros of home delivery of medication service, and (5) statements to evaluate their perception toward the cons of this service. The responses for the perception part of the survey (parts 3–5), were assessed using the 5-Likert scale as follows “5: strongly agree,” “4: agree,” “3: neutral,” “2: disagree,” or “1: strongly disagree.” Cronbach's α measure was calculated to assess the internal consistency for each the three perception sections (Sections Results, Discussion, and Conclusion). Values of 0.830, 0.865, and 0.951 were obtained for the third, fourth and fifth domains, respectively. This indicates an acceptable internal consistency (9).

The study survey was then uploaded on the Google Form platform, and it was distributed through Facebook to target Jordanian community pharmacists' groups on Facebook. At the beginning of the survey, pharmacists were provided with a brief description of the study objectives and what is expected from their participation including the time needed to fill the survey (10 min), the anonymity of their participation was confirmed, and they were also informed that their participation is voluntary. They were then asked to electronically approve and provide their consent before filling out the study survey.

### Sample size calculation

The minimum sample size was calculated using the statistical formula of Fisher for calculating sample size: *n* = P × (1–P) × z^2^/d^2^. Using the most conservative proportion of pharmacists willing to use this service (*P* = 50%), a confidence level of 0.95 (*z* = 1.96), and a desired precision (d) of 5%, the minimum sample size was found to be 385 pharmacists.

### Statistical analyses

Statistical analyses were conducted using SPSS version 22 (SPSS Inc., Chicago, IL, USA). Descriptive statistics were used to describe the data. Categorical variables were presented as frequencies and percentages, while continuous variables were reported as median and interquartile range (IQR). Normality was checked using the Shapiro-Wilk test, with a *P* > 0.05 indicates normally distributed variables.

Logistic regression analysis was carried out to screen for independent factors associated with pharmacists' willingness to use this service. Following simple logistic regression, any variable with a *P* < 0.250 was considered eligible for entry in multiple logistic regression analysis. Before conducting multiple logistic regression analysis, variables were checked for the absence of multicollinearity (i.e., Pearson correlation coefficient <0.9 any two variables). A *P* ≤ 0.05 was considered statistically significant to identify factors associated with pharmacists' willingness to use this service.

Regarding the internal consistency of the study survey, a Cronbach alpha value > 0.7 was considered to indicate an acceptable internal consistency (9).

## Results

In this study, 324 community pharmacists agreed to participate in the study and filled-out the study questionnaire. The median age of the respondent pharmacists was 34.0 years (IQR = 11), with more than half of them being females (*n* = 193, 59.6%). More than three-quarters of the pharmacist (*n* = 248, 76.5%) had undergraduate degree (BPharm or Pharm D), and more than half of them (*n* = 172, 53.1%) were working in chain community pharmacies. Pharmacists were recruited from Amman the capital of Jordan (*n* = 172, 53.1%) and other governorates (*n* = 152, 46.9%). Pharmacists had a median of 6 years of experience (IQR = 9) as community pharmacists, and around 60% of them (*n* = 193, 59.6%) serve 50 patients or more per day. For more information about the socio-demographic characteristics of the study respondents, refer to Table 1.

**Table 1:** Socio-Demographic characteristics of the study respondent (*n* = 324).

**Parameter**	**Median (IQR)**	***n* (%)**
**Age (years)**	34.0 (11.0)	
**Gender**
Females		193 (59.6)
Males		131 (40.4)
**Educational level**
Undergraduate level (BPharm or Pharm D)		248 (76.5)
Postgraduate level (M.Sc. or Ph.D.)		76 (23.5)
**Job status**
Owner		101 (31.2)
Employee		223(68.8)
Experience as community pharmacists (years)	6.0 (9.0)	
**Site of work**
Independent community pharmacy		152 (46.9)
Chain community pharmacy		172 (53.1)
**Location of pharmacy**
Amman		172 (53.1)
Others		152 (46.9)
**Number of patients you service/day**
<50		131 (40.4)
≥50		193 (59.6)

IQR, interquartile range.

Regarding pharmacists' awareness of the home delivery of medication service (Table 2), the majority of pharmacists (*n* = 303, 93.5%) revealed that they had heard about the service, and around three-quarters of them (*n* = 244, 75.3%) reported to be willing to use the service at their community pharmacies. Moreover, most of the pharmacists (*n* = 274, 84.6%) believed that the introduction of home delivery of medication service would make pharmacy service more efficient.

**Table 2:** Pharmacists' awareness, opinion, and willingness to use home delivery of medications (*n* = 324).

**Parameter**	***n* (%)**
**Did you hear about home delivery of medication service?**
No	21 (6.5)
Yes	303 (93.5)
**Do you feel that the introduction of home delivery of medication service**	
**makes your pharmacy more efficient?**	
No	50 (15.4)
Yes	274 (84.6)
**Are you willing to use home delivery of medication service?**
No	80 (24.7)
Yes	244 (75.3)

When asked about the differences between home delivery of medications and in-store drug refill (Table 3), around two-thirds of the pharmacists (*n* = 210, 64.8 %) found to believe that they might be less available to answer questions using home delivery of medications compared to that of in-store refill, and nearly half of them (*n* = 163, 50.3%) thought that they cannot explain important points about prescription while providing home delivery of medications compared to that in-store refill. Moreover, less than half of the pharmacists agreed or strongly agreed that unlike in-store service, home delivery of medications is suitable only for OTC but not for prescriptions medications (*n* = 129, 39.8%), and only for refill prescriptions but not for new prescriptions (*n* = 153, 47.2%).

**Table 3:** Pharmacists perceived difference between home delivery of medications and in-store drug refill (*n* = 324).

**Statement**	**Agreed/strongly agreed *n* (%)**
Pharmacist might be less available to answer questions using home delivery of medications compared to that of in-store refill	210 (64.8)
Written counseling information might be less supplied using home delivery of medications compared to in-store refill	142 (43.8)
Pharmacist cannot explain important points about prescription while providing home delivery of medications compared to that in-store refill	163 (50.3)
Unlike in-store service, home delivery of medications is suitable only for refill prescription but not for new prescription	153 (47.2)
Unlike in-store service, home delivery of medications is suitable only for OTC but not for prescriptions medications	129 (39.8)

OTC, over the counter.

**Figure 1:**
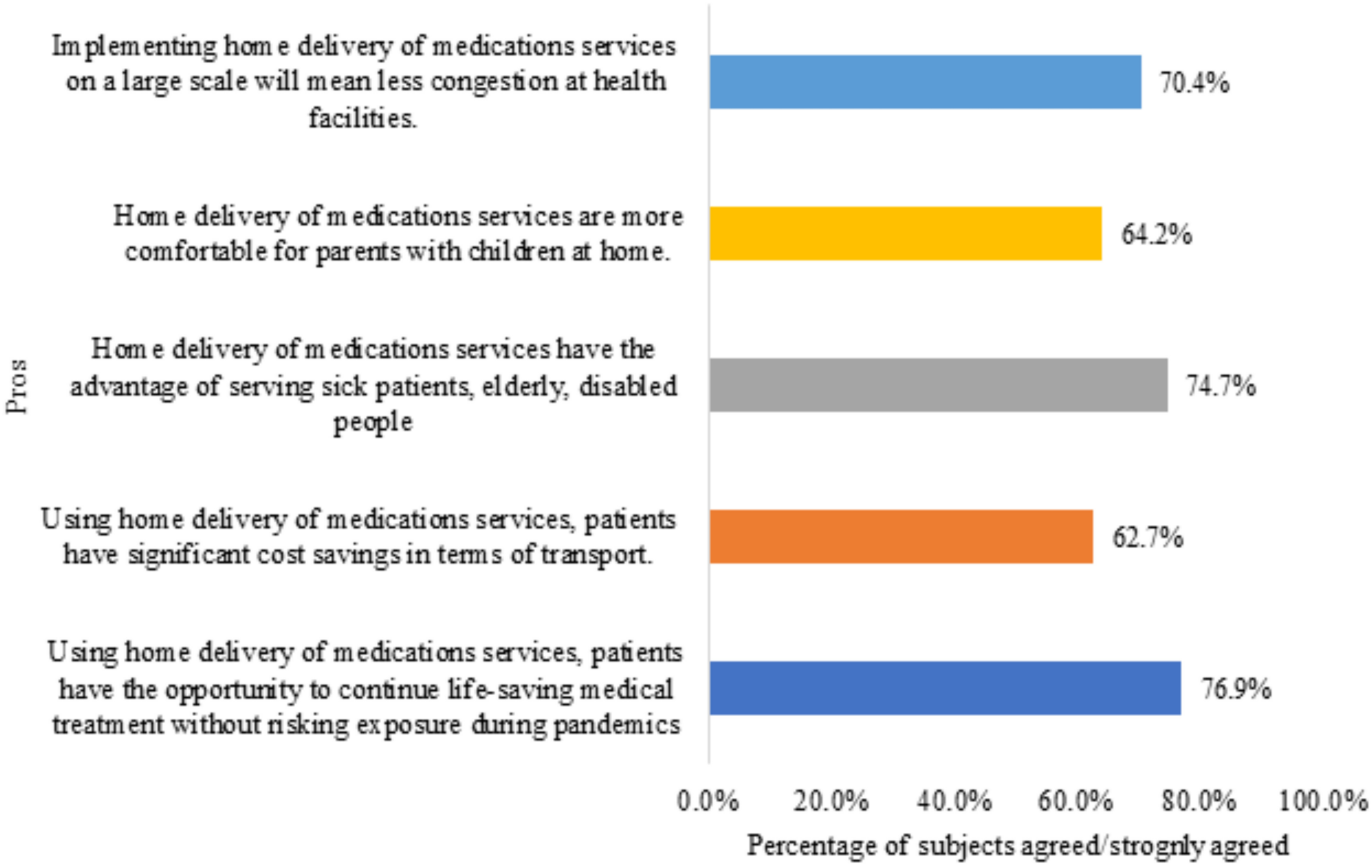
Participants' perception of the pros of home delivery of medication service (*n* = 324).

Regarding the pros of home delivery of medications (Figure 1), more than half of the pharmacists believed that home delivery of medications service has the opportunity to continue life-saving medical treatment without risking exposure during pandemics (*n* = 249, 76.9%), serving sick patients, elderly, disabled people (*n* = 241, 74.4%), decrease congestion at health facilities (*n* = 228, 70.4%), is more conformable for parents with children at home (*n* = 208, 64.2%), and have cost saving in term of transport (*n* = 203, 62.7%).

**Figure 2:**
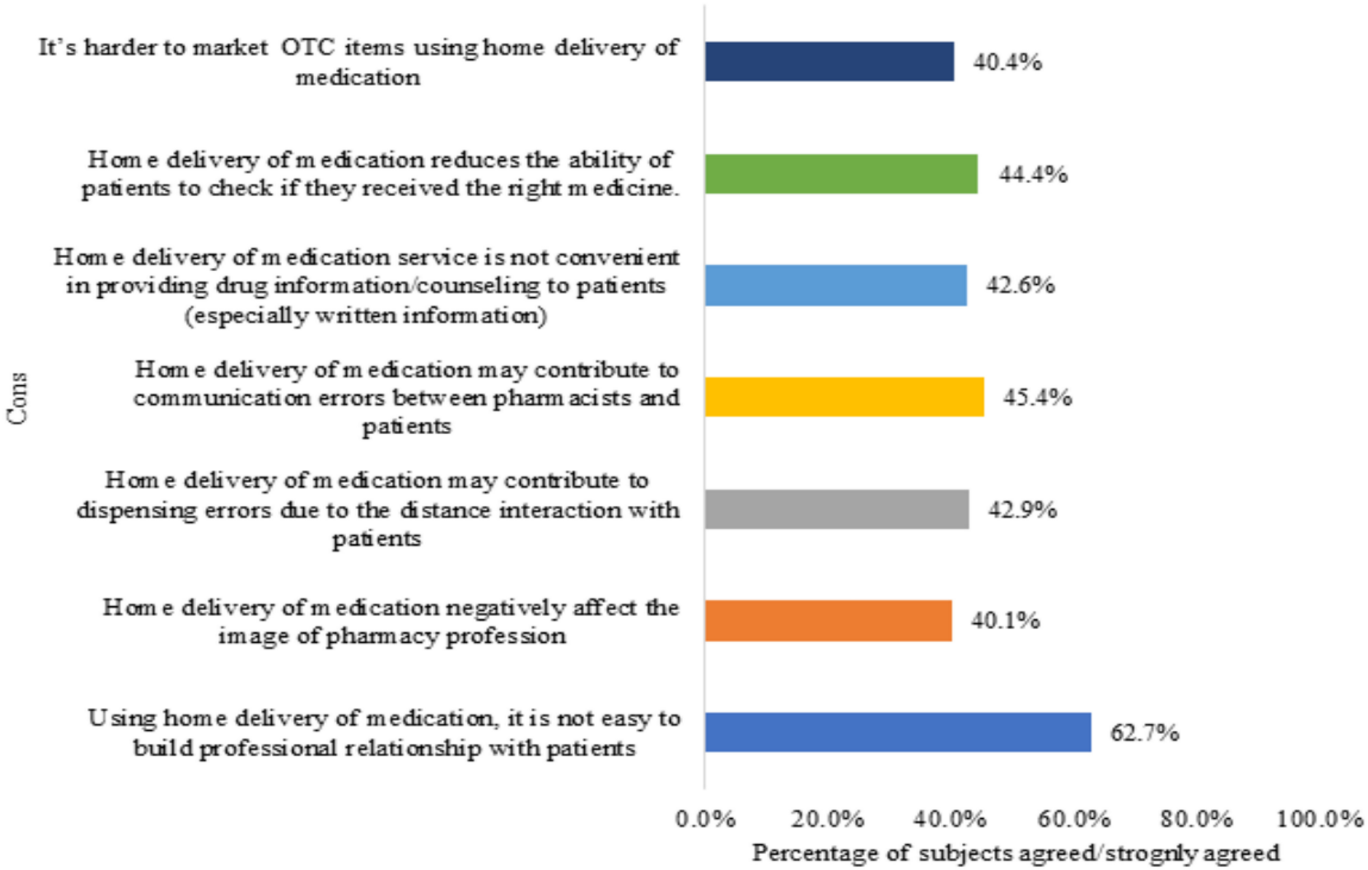
Participants' perception of the cons of home delivery of medication service (*n* = 324).

On the other hand, the perception of pharmacists regarding the cons of home delivery of medication service was also assessed (Figure 2). As seen in Figure 2, around two-third of the pharmacists (*n* = 203, 62.7%) strongly agreed/agreed that by using home delivery of medication it is not easy to build professional relationship with patients. Also, <50% of the pharmacists believed that home delivery of medication may contribute to communication errors between pharmacists and patients (*n* = 147, 45.4%), reduce the ability of patients to check if they received the correct medications (*n* = 144, 44.4%), and contributes to dispensing errors (*n* = 139, 42.9%). Other cons of this service are presented in Figure 2.

Finally, logistic regression analysis was performed to assess predictors affecting pharmacists' willingness to use home delivery of medication service (Table 4), and results showed that those pharmacists serving 50 patients or more per day were more willing to use the service than those serving <50 patients per day (OR = 2.058, *P* = 0.032). The logistic regression model was fit well, ${\text{χ}}_{\left(8\right)}^{2}$ = 8.149, *P* = 0.419 (Hosmer-Lemeshow goodness-of-fit). The model explained 10.3% (Nagelkerke *R*^2^) of pharmacists' willingness to use home delivery of medication service and correctly classified 80.4% of cases.

**Table 4:** Assessment of factors associated with pharmacists' willingness to use home delivery of medications service (*n* = 324).

**Parameter**	**Willingness to use home delivery of medication service [0: Rejecter, 1: Acceptor]**			
	**OR**	***P*-value#**	**OR**	***P*-value$**
Age (years)	1.009	0.565	–	–
**Gender**
Females	Reference	0.162^∧^	1.442	0.295
Males	1.458			
**Educational level**
Undergraduate level (BPharm or Pharm D)	Reference	0.497	–	–
Postgraduate level (M.Sc. or Ph.D.)	0.818			
**Job status**
Owner	Reference	0.794	–	–
Employee	0.929			
Experience as community pharmacists (years)	1.078	0.012^∧^	1.052	0.084
**Site of work**
Independent community pharmacy	Reference	0.015^∧^	1.561	0.179
Chain community pharmacy	1.885			
**Location of pharmacy**
Amman	Reference	0.693	–	–
Others	1.108			
**Number of patients you service/day**
<50	Reference	0.046^∧^	2.058	0.032^*^
≥50	1.681			

#Using simple logistic regression, $using multiple logistic regression,

∧ eligible for entry in multiple logistic regression,

* significant at 0.05 significance level.

## Discussion

In this study, we investigated pharmacists' perception of home delivery of medications service in Jordan and their willingness to use the service. We also explored factors associated with pharmacists' willingness to use the service. More than 75% of participants are willing to use this service, while most of them believed that home delivery service would be effective at many levels. This could be explained by the fact that community pharmacists are overwhelmed with many duties, this might relieve the burden, bypassing the need to visit a retail pharmacy. Moreover, this will be more convenient, accessible, and affordable particularly for patients with special needs. However, an important fact that this study revealed is that a high proportion of these experienced pharmacists believe that much important information may be skipped if medications are not dispensed face to face at the pharmacy site. This could be particularly true in patients on complex regimens, such as anticoagulants, insulin therapy, and antiepileptic drugs (10). In light of these findings, integration of telehealth services among home delivery services would be extremely important in covering this missing aspect. The trends for telehealth services provided by pharmacists may include live videos for demonstrations of inhalers, injectables, nasal sprays, patches or complex regimens, or audio-only visits for counseling on major drug information patients should know (11). A complete medication review in conjunction with medication dispense when the encounter is telephonic. This sincere novel practice may ensure pharmacist-patient interaction, decreasing the risk for medication errors including adverse events, and medication misuse. However, the lack of financial incentives in recognition to these additional duties may be an obstacle. This also highlight the urge to reconsider a financial remuneration for community pharmacists to encourage the tele system technology in counseling patients about their medicines (12).

The current study also reported that pharmacists recognize that the most advantageous groups for this service are patients on life-saving and chronic medications and elderly patients. A recent study found that patients on maintenance medications through home delivery are more likely to take them as prescribed for conditions like diabetes, hypertension, and dyslipidemia (13). However, previous studies that investigated the benefits of home delivery services were self-administered questionnaires susceptible to social desirability bias (10, 12, 14–17). Therefore, there is a need for studies with a stronger methodological rigor to thoroughly address this regard.

Despite the fact that the implementation of home delivery of medications is associated with several pros, such as treatment continuity, economic savings, decongestion of the healthcare centers, avoiding traveling expenses, saving patients' time, and enhancing the quality of life (10, 17), there are potential drawbacks that should be highlighted. First, implementing a home drug delivery service without integrating it with the telepharmacy system, compromises the optimal provision of the pharmaceutical care (2). Second, home drug delivery services might be accompanied by a risk of breaching patients' confidentiality, especially those suffering from medical conditions with a greater social stigma or disability (17). It is noteworthy, that in Jordan, a Middle Eastern developing country, that is experiencing an economic meltdown, implementing home drug delivery is still lagging behind (18). This service is associated with serious limitations in its development and application, especially in terms of the absence of a national legal framework concerning remote medication dispensing and informed delivery (2). In addition, the concept of telepharmacy and the use of technology for remote clinical assistance is not yet established, where Jordanian community pharmacists do not have access to patients' electronic medical records, which might jeopardize patient care provision and increase the risk of medication errors. Therefore, the implementation of a home drug delivery service should be incorporated into a strategic plan for executing remote medical provision.

In addition, pharmacists serving more patients per day were found more willing to use the service than those serving less patients in this study. This could be explained that implementing home delivery of medications may contribute in decreasing congestion at the community pharmacies and thus, protect people from infection, yet meeting patients' demands (19).

During the COVID-19 pandemic, pharmaceutical care services were rapidly remodeled beyond the scope of the traditional practice (11). Community pharmacists were securing patients' medications in the comfort of their homes, thus, contributing to combating transmission rates while ensuring better medication adherence for vulnerable patients that are more prone to fall behind prescriptions (15). Before the pandemic, this practice was not that common, public, or legal in many developing countries that are still fighting to establish and adopt the pharmacy practice field's modern role (11, 20).

The contemporary pharmaceutical care provider follows a patient-centered approach that aims to achieve individualized health-related outcomes (21, 22). While tackling a patient-centered approach, considerations should be made particularly in vulnerable sick and disabled patients, the elderly, and many other patients where a visit to the pharmacy is not a simple task (14, 20). While during the pandemic several studies were investigating the implementation of home delivery, and other telehealth services, they were all conducted from patients' perspectives, and none of these studies the perception of community pharmacists toward this practice (10, 14–17). Investigating community pharmacists' perception and willingness of adopting this service is necessary to ensure their effective involvement and awareness of the pros and cons of this service. We believe that data from such studies can be essential in directing authorities involved in adopting telepharmacy and designing a home-delivery service scheme at a national level in Jordan.

This study was proactive in investigating the pharmacists' willingness to implement home drug delivery services, however, it was associated with several limitations. First, this observational study only reflected the pharmacists' intention to apply a new service, without investigating its actual practice in some Jordanian pharmacies, noting that there is an absence of a clear national declaration that permits remote medication dispensing. Second, this study did not examine the community pharmacies' capabilities in implementing remote medication dispensing and the factors that might hinder its optimal execution. Third, the sample size recruited in this study was lower than the minimum calculated sample size. Finally, the current study did not assess the associated drug dispensing errors. Therefore, it would be interesting to conduct long-term studies to determine the employment of remote drug dispensing embedded within a telepharmacy model.

## Conclusion

This study indicated that most pharmacists are willing to use the service at their community pharmacies, especially those serving more patients per day. However, a national framework should be launched to organize and assist in the introduction and conduction of this remote health service in Jordan since no regulations are in place yet. Moreover, the medication home delivery model should be integrated with telepharmacy, to preserve all the pharmaceutical care benefits and to decrease the drawbacks of this service.

## Data availability statement

The original contributions presented in the study are included in the article, further inquiries can be directed to the corresponding authors.

## Ethics statement

The study was granted ethical approval by the Institutional Board Committee at Applied Science Private University (Approval number 2022-PHA-4). The participants provided their written informed consent to participate in this study.

## Author contributions

All authors listed have made a substantial, direct, and intellectual contribution to the work and approved it for publication.

## Conflict of interest

The authors declare that the research was conducted in the absence of any commercial or financial relationships that could be construed as a potential conflict of interest.

## Publisher's note

All claims expressed in this article are solely those of the authors and do not necessarily represent those of their affiliated organizations, or those of the publisher, the editors and the reviewers. Any product that may be evaluated in this article, or claim that may be made by its manufacturer, is not guaranteed or endorsed by the publisher.
